# The Effect of Promoter and RBS Combination on the Growth and Glycogen Productivity of Sodium-Dependent Bicarbonate Transporter (SbtA) Overexpressing *Synechococcus* sp. PCC 7002 Cells

**DOI:** 10.3389/fmicb.2021.607411

**Published:** 2021-04-13

**Authors:** Jai Kumar Gupta, Shireesh Srivastava

**Affiliations:** ^1^Systems Biology for Biofuels Group, International Centre for Genetic Engineering and Biotechnology (ICGEB), New Delhi, India; ^2^Department of Biotechnology-International Centre for Genetic Engineering and Biotechnology (DBT-ICGEB), Centre for Advanced Bioenergy Research, New Delhi, India

**Keywords:** promoter, cyanobacteria, biomass, feedstock, glycogen stores, bicarbonate transport, RBS

## Abstract

Sodium dependent bicarbonate transporter, SbtA is a high-affinity, inducible bicarbonate transporter in cyanobacterial cells. Our previous work has shown that overexpression of this transporter can significantly increase growth and glycogen accumulation in *Synechococcus* sp. PCC 7002 cells. In this work, we have tested the effect of two different RBS sequences (RBS1: GGAGGA and RBS2: AGGAGA) and three different promoters (P_cpcB_, P_cpcB__560_, and P_rbcL__2_) on the growth and glycogen production in SbtA-overexpressing *Synechococcus* sp. PCC 7002 cells. Our results show that P_cpcB_ or P_cpcB__560_ were more effective than P_rbcL__2_ in increasing the growth and glycogen content. The choice of RBS sequence had relatively minor effect, though RBS2 was more effective than RBS1. The transformant E, with P_cpcB__560_ and RBS2, showed the highest growth. The biomass after 5 days of growth on air or 1% CO_2_ was increased by about 90% in the strain E compared to PCC 7002 cells. All transformants overexpressing SbtA had higher glycogen content. However, growing the cells with bubbling of 1% CO_2_ did not increase cellular glycogen content any further. The strain E had about 80% higher glycogen content compared to WT PCC 7002 cells. Therefore, the glycogen productivity of the strain E grown with air-bubbling was about 2.5-fold that of the WT PCC 7002 cells grown similarly. Additionally, some of the transformants had higher chlorophyll content while all the transformants had higher carotenoid content compared to the PCC 7002 cells, suggesting interaction between carbon transport and pigment levels. Thus, this work shows that the choice of photosynthetic promoters and RBSs sequences can impact growth and glycogen accumulation in SbtA-overexpressing cells.

## Introduction

Cyanobacteria are fast-growing prokaryotic microorganisms capable of oxygenic photosynthesis. They offer advantages of small genome size and genetic amenability (Gantt, 2011; Nozzi et al., 2013; Singh et al., 2016; Jaiswal et al., 2018; Ahmad et al., 2020) and serve as attractive hosts for synthetic biology applications, ranging from metabolic engineering for the production of industrial biochemicals to microbial energy storage (Englund et al., 2016; Vavitsas et al., 2019; Sengupta et al., 2020b). Genetic engineering approaches and tools have been developed for some species (Huang and Lindblad, 2013). Many cyanobacteria, including all the model species, are naturally transformable and can integrate transgenes into their chromosomes by homologous recombination (Ludwig and Bryant, 2012; Vavitsas et al., 2019; Sengupta S. et al., 2020). Cultivation of marine cyanobacteria would help in large scale sustainable conversion of greenhouse CO_2_ to useful products while reducing freshwater usage (Markley et al., 2015; Englund et al., 2016).

*Synechococcus* sp. PCC 7002 (hereafter PCC 7002) is a fast-growing marine cyanobacterium with an optimal growth temperature of 38°C (Ludwig and Bryant, 2012), and is tolerant to variations in nutrient availability, salinity, pH, irradiance and temperature (Ludwig and Bryant, 2012). PCC 7002 has been engineered earlier for the synthesis of a number of bioproducts viz. ethanol (Kopka et al., 2017), succinic acid, bioplastics, terpenoids (Davies et al., 2014). However, the growth rate and cell titers are still low to be commercially viable (Markley et al., 2015). We have previously engineered PCC 7002 with the bicarbonate transporters SbtA and BicA and shown improvement in their growth and intracellular product (glycogen) content (Gupta et al., 2020). While BicA is a relatively well-studied bicarbonate transporter, there are only a few studies on SbtA. SbtA is a high-affinity, low flux, inducible sodium (Na +) dependent HCO_3_^–^ transporter, functioning as an Na^+^/HCO_3_^–^ symporter, that has been shown to be upregulated in carbon limiting conditions in earlier studies (Shibata et al., 2002; Price, 2011). Our work had shown that overexpression of SbtA can promote the growth of PCC 7002 cells to the same levels as that observed with the overexpression of BicA (Gupta et al., 2020).

Protein levels are commonly controlled through regulation of transcription and translation initiation rates (Markley et al., 2015). In previous studies it has been shown that promoter and ribosome binding sites (RBS) sequences greatly affect expression of a target gene (Shi et al., 2018). Promoters are one of the key synthetic biology components in cyanobacteria. Transcription rates can be regulated by choosing a suitable promoter for a target gene (Markley et al., 2015). Many promoters—constitutive, inducible and repressible are available (Vavitsas et al., 2019). Strength of a promoter shows a direct relationship with the gene expression (Bienick et al., 2014). Activity of promoters can vary based on culture conditions and presence of metal ions (Englund et al., 2016). The promoters P_rbcL__2A_ and P_cpcB_ are responsible for the transcription of genes coding for large subunit 2A of RuBisCO and c-phycocyanin beta subunit, respectively (Huang et al., 2010; Markley et al., 2015). These are among the most common promoters used in cyanobacteria (Till et al., 2020). Presence of multiple transcription factor binding sites (TFBSs) is crucial for the promoter strength. P_cpcB__560_, a modified version of *Synechocystis* sp. PCC 6803 *cpcB* promoter, was reported in an earlier study and contains the P_cpcB_ promoter with 14 TFBSs (Zhou et al., 2014). The promoter size of P_cpcB__560_ was smaller than the native promoter P_cpcB_ by 29 bps. The expression of heterologous genes using the P_cpcB__560_ promoter in *Synechocystis* sp. PCC 6803 cells resulted in expression level comparable to that obtained in *Escherichia coli* (*E. coli*) (Zhou et al., 2014).

Another element that can significantly impact the translation rate and hence the protein levels is the strength or accessibility of ribosome-binding sites (RBS) (Reeve et al., 2014; Markley et al., 2015).

In the present study we have investigated the combinatorial effect of three strong promoters (viz. P_rbcL__2A_, P_cpcB_, and P_cpcB__560_, all from *Synechocystis* sp. PCC 6803) and two RBSs, of different strengths, viz. RBS1 (GGAGGA) and RBS2 (AGGAGA), on the expression of a membrane protein, SbtA, and the associated physiological responses (cellular growth and glycogen content) in the PCC 7002 cells. Our results indicate that the appropriate combination of strong promoter and RBS can indeed impact SbtA expression, growth and glycogen levels.

## Materials and Methods

### Materials

Ampicillin disodium salt, KCl, bacteriological agar and kanamycin monosulfate were purchased from Amresco (Solon, OH, United States). Tris base and NaCl were from Thermo Fisher Scientific (Mumbai, India) while CuSO_4_⋅5H_2_O, H_3_BO_3_, KH_2_PO_4_, Na_2_EDTA.2H_2_O, CaCl_2_⋅2H_2_O, MgSO_4_⋅7H_2_O, NaNO_3_, FeCl_3_⋅6H_2_O, CoCl_2_⋅6H_2_O MnCl_2_⋅4H_2_O, ZnCl_2_, vitamin B_12_, and RNaseZAP were from Sigma-Aldrich (St. Louis, MO, United States). Primers were purchased from Sigma Aldrich. Details of kits used in various molecular biological methods such as genomic DNA and plasmid isolation, PCR purification etc. are mentioned further. All the restriction enzymes used in the study were from Thermo-Scientific (Mumbai, India).

### Cyanobacterial Strains and Growth Conditions

*Synechococcus* sp. PCC 7002 cells, originally obtained from the Pasteur Culture Collection (PCC), Paris, France, were cultured in A^+^ medium in 250 mL shake flasks (Ludwig and Bryant, 2012) in an incubator shaker (Innova 44R, New Brunswick Scientific, Edison, NJ, US) at 150 rpm and 30°C with a light:dark cycle of 16:8 h under a light intensity of 150 μmol m^–2^ s^–1^, illuminated by LED lamps. The composition of medium A^+^ is 18 g L^–1^ NaCl, 5.0 g L^–1^ MgSO_4_⋅7H_2_O, 30 mg L^–1^ Na_2_ EDTA⋅2H_2_O, 0.6 g L^–1^ KCl, 0.37 g L^–1^ CaCl_2_, 1.0 g L^–1^ NaNO_3_, 50 mg L^–1^ KH_2_PO_4_, 10 mL L^–1^ Tris/HCl (Stock solution 100 g L^–1^, pH 8.2), 1 mL L^–1^ A + trace components. 1 mL L^–1^ vitamin B_12_ (stock solution 4 mg L^–1^) was added. A+ trace components (1000X) composition includes: 34.3 g L^–1^ H_3_BO_3_, 0.315 g L^–1^ ZnCl_2_, 30 mg L^–1^ MoO_3_ (85%), 0.389 g L^–1^ FeCl_3_⋅6H_2_O, 0.43 g L^–1^ MnCl_2_⋅4H_2_O, 0.3 mg L^–1^ CuSO_4_⋅5H_2_O, 1.2 mg L^–1^ CoCl_2_⋅6H_2_O (Utex Culture Collection of Algae, 2021). Experiments were conducted in 250 mL Dreschel (gas-washing) bottles (Borosil, India) containing 150 mL culture. The cultures were bubbled with air or 1% CO_2_ in air through a gas dispersion tube with porous fritted glass tip (Sigma Aldrich Chemical Co.) at the rate of 0.5 L/min. The culture bottles were kept in a water bath shaker maintained at an optimal temperature of 38°C (Ludwig and Bryant, 2012) and continuously illuminated from the top with LED lights having a light intensity of 350 μmol m^–2^ s^–1^. Biomass density was monitored every 24 h by measuring the optical density at 730 nm (OD_730_). Cultures were stopped at the end of 5 days for quantification of various parameters.

### Plasmids Construction and Cloning

**FIGURE 1:**
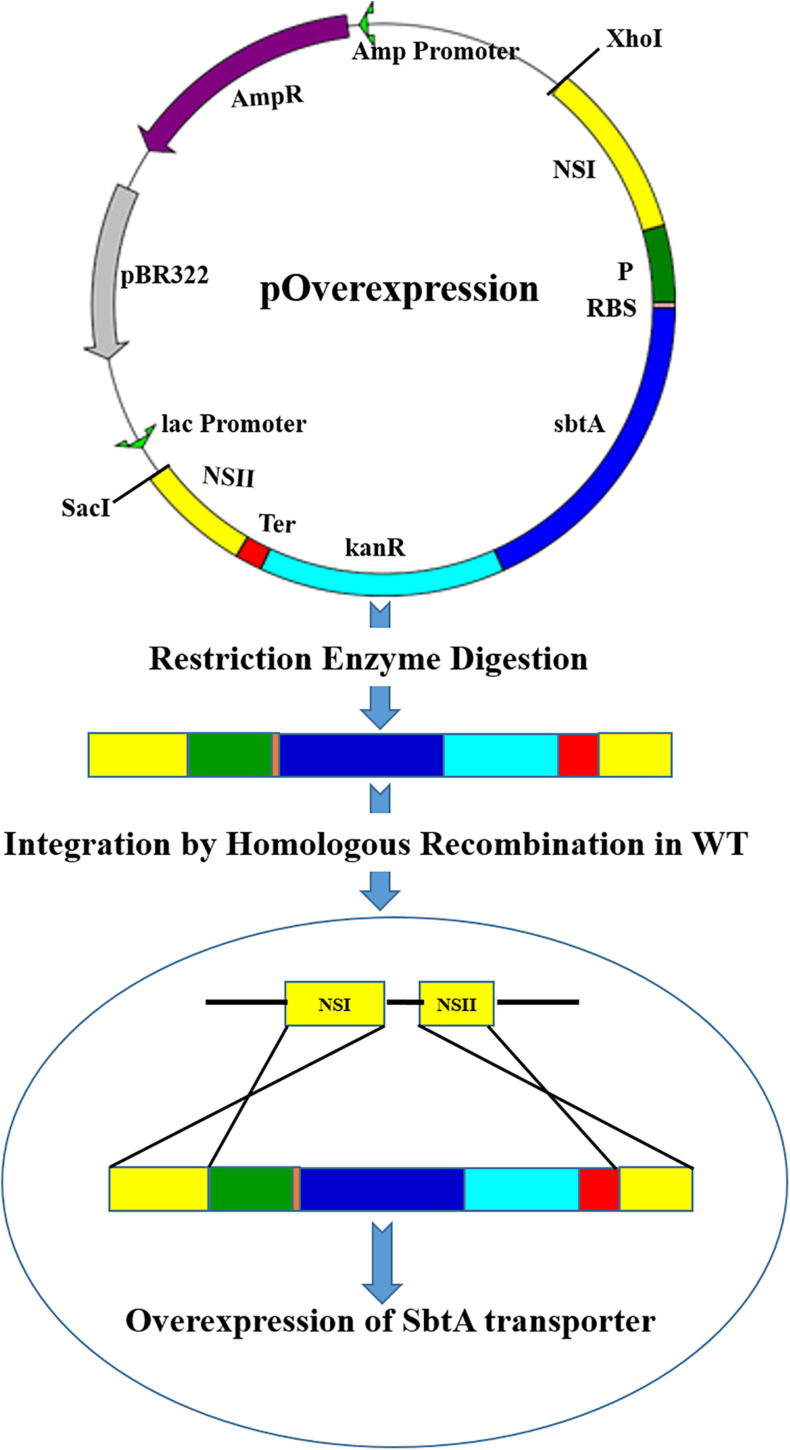
A diagram showing integration of the gene cassettes into the genomic DNA of PCC 7002 through homologous recombination. The vector pOverexpression shows a generalized plasmid for the overexpression of SbtA with different promoters and RBSs sequences. NSI (neutral site I), P (Promoter), RBS (ribosome binding site), *sbtA* (gene coding for sodium dependent bicarbonate transporter SbtA), kanR (kanamycin resistance gene), Ter (terminator), and NSII (neutral site II).

The promoters used in this study- P_cpcB_, P_cpcB__560_ and P_rbcL__2A_, were amplified from the genomic DNA of *Synechocystis* sp. PCC 6803 while the kanR2 gene was amplified from the commercial vector pET-28a(+). The genes *sbtA* (SYNPCC7002_A0470) encoding the HCO_3_^–^ transporter (Price et al., 2011), neutral sites NS1 (SYNPCC7002_A0935) and NS2 (SYNPCC7002_A0936) (Davies et al., 2014) encoding hypothetical proteins and GroEL terminator were amplified from the genomic DNA of PCC 7002 cells. The DNA fragments cloned are shown in Figure 1. These DNA sequences were PCR amplified using Phusion polymerase (Thermo Scientific, Mumbai, India). GeneJET PCR purification kit (Thermo Fisher Scientific, Lithuania) was used for purification of the PCR-amplified products. The RBS sequences and an extra 7 bp sequence for ribosome binding were included in the forward primers designed for the *sbtA* gene (see Supplementary Table 2 for the list of primers used in this study). The promoters stretch used in this work did not include the native RBS sequence. pBluescript II SK(+) was used as a cloning vector for the ligation of DNA fragments by sticky-end cloning, performed using the specific restriction enzymes and T4 DNA ligase. pBluescript II SK(+) was selected as a cloning vector because of its small backbone size (2961 base pairs) and presence of a large number of common restriction enzyme recognition sites in its MCS (multiple cloning site) region (Alting-Mees and Short, 1989; Abe et al., 1996; Hong et al., 2009). The cloning was performed in *E. coli* DH-5α as follows. Competent cells were prepared using a previously-published protocol (Im, 2011). Transformation of the competent *E. coli* cells was done as per an earlier published protocol (Froger and Hall, 2007) with some modifications. A vial containing 50 μL stock of competent cells, kept at −80°C was thawed on ice. Three to five microliter of the ligation mixture was added to the competent cells. The tube was flicked 3–4 times to mix the cells and DNA. Cells were further kept on ice for 30 min. Heat shock was given at 42°C for 90 s, following which the tube was quickly kept on ice for 2 min. Eight hundred microliter of sterile LB broth (Himedia, India) was added and the tube was kept in a 37°C incubator shaker at 150 rpm for 1 h. Cells were then centrifuged at 4,000 × *g* for 5 min at room temperature. Eight hundred microliter of the supernatant was discarded and the pellet was resuspended and spread on a LB agar plate containing 100 μg/mL ampicillin, and incubated overnight (~16 h) at 37°C. Single colonies obtained were used for further analyses. After transformation with the respective plasmids, the *E. coli* cells were cultured overnight at 37°C in glass tubes containing 5 mL LB medium supplemented with the appropriate antibiotic concentration at 150 rpm in an incubator shaker (Innova 44R, New Brunswick Scientific, NJ). For the extraction of plasmids, the GeneJET Plasmid Miniprep Kit (Thermo Fisher Scientific, Vilnius, Lithuania) was used.

Two microgram g of the cloned plasmids, viz. pA, pB, pC, pD, pE, and pF (Supplementary Figure 4) were digested with the restriction enzymes *Xho*I and *Sac*I for 30 min in a 1.5 mL tube with 30 μL reaction mixture at 37°C in a water bath to separate the cassette of interest from the host vector. The digested vector was separated on a 1% agarose gel and the DNA of interest was eluted in MilliQ water using a gel extraction kit (Thermo Scientific, Vilnius, Lithuania). PCC 7002 cells were transformed with the resulting DNA solution as per the following protocol.

### Transformation of *Synechococcus* sp. PCC 7002 Cells

The work was approved by the institutional biosafety committee (IBSC). Transformation was based on the earlier protocols (Ruffing, 2014). Linearized DNA fragments obtained from DNA extracted from respective transformed *E. coli* host cells were used for the transformation of PCC 7002. PCC 7002 cells were grown in A^+^ medium under 150 μmol m^–2^ s^–1^ in incubator shaker (Innova 44R, New Brunswick Scientific) at 30°C with 150 rpm until an OD_730_ ∼ 1. Eight hundred microliter culture was taken in a microcentrifuge tube and the DNA (∼ 1 μg) was added. The tube was kept for 24 h in the incubator shaker and then centrifuged at room temperature for 5 min at 2,500 × *g*. The pellet obtained was spread on A^+^ agar plates containing 50 μg/mL kanamycin monosulfate. The single isolated colonies were re-streaked four more times on antibiotic plates for complete segregation of transformants. Genomic DNA of the colonies was extracted and PCR was done to confirm integration of the constructs in the genome. Positive clones were used for further analyses.

### Analysis of Gene Expression by Real-Time PCR and Western Blotting

RNA and proteins were extracted as in our earlier study (Gupta et al., 2020). For RNA isolation, 50 mL culture in exponential phase was centrifuged for 5 min at 4000 × *g*. The pellet was crushed in presence of liquid nitrogen using a pestle wiped with RNaseZAP and washed with RNase-free water. RNeasy Plant Mini Kit (Qiagen, Hilden, Germany) was used as per the kit’s protocol to isolate RNA. The resulting RNA was treated with DNase-I to remove any traces of DNA. cDNA was synthesized from 1 μg of the DNase-treated RNA using a cDNA synthesis kit (Thermo Fisher Scientific, Lithuania). Quantitative real time PCR (qRT-PCR) was conducted using KAPA SYBR Fast Kit (Kapa Biosystems, Sigma-Aldrich, South Africa). An earlier study, that had compared suitability of six candidate genes to be used as internal reference gene in PCC 7002 cells, reported phosphoenolpyruvate carboxylase (SYNPCC7002_A1414, *ppC*), as a suitable reference gene on the basis of its M-value (gene stability measure) (Szekeres et al., 2014). Therefore, in this study, *ppc* was used as the internal reference gene. Primers used in qRT-PCR are listed in Supplementary Table 4). 2^(–ΔΔ*Ct)*^ values were calculated for the relative change in expression.

An earlier published protocol was used (Gupta et al., 2020) for western blotting. 20 mL culture was centrifuged and the pellet collected. 1mL lysis buffer (Du et al., 2014) and 100 μL of 0.1 mm glass beads were added and the cells were lysed using a beadbeater (FastPrep-24, MP Biomedicals, India) for 10 cycles of 30 s each with alternate chilling in between. The resulting cell lysate was centrifuged at 4°C for 15 s at 21,000 × *g*; the supernatant was transferred to another tube and centrifuged for 10 min. The pellet obtained was supplemented with sodium dodecyl sulfate (SDS) sample buffer containing 4% (w/v) SDS, 10% glycerol, 1 mM dithiothreitol (DTT) and 62.5 mM Tris–HCl, pH 6.8. Samples were incubated for 20 min at 70°C, and centrifuged for 15 min at 21,000 × *g*. Supernatant fraction was collected and protein concentration measured with a Pierce BCA Protein Assay Kit (Thermo Fisher Scientific, Rockford, United States). 100 μg protein sample was loaded and separated on a 12% SDS-polyacrylamide gel. Proteins were transferred onto a 0.22 μm PVDF membrane (Bio-Rad, United States) using a semi-dry transfer apparatus (Bio-Rad, United States) as per the company’s protocol. The expression of His6-tagged SbtA was detected with a monoclonal anti-His antibody (Sigma-Aldrich) and a horseradish peroxidase-conjugated secondary antibody. Proteins were stained using a DAB substrate kit (Thermo Fisher Scientific, Rockford, United States) and visualized using chemiluminescence.

### Dry Cell Weight Determination

The dry cell weights were measured as per our previously published protocol (Gupta et al., 2020). After centrifuging the cell culture (50 mL) at 2,500 × *g* for 10 min, the pellet obtained was washed twice in 8.25 mM Tris–HCl buffer (pH 8.2). Then, the pellet was dried for approximately 36 h at 65°C until the weight of the tube became constant. The weight of the tube was subtracted from the total weight to calculate the dry cell weights.

### Measurement of Glycogen Content

Glycogen content was determined as per the earlier published literature (Dutt and Srivastava, 2018; Gupta et al., 2020). Cells were resuspended to reach OD_730_ of ∼2. 1 mL of the culture was pelleted down at room temperature for 5 min at 4,000 × *g*, and 7.5% H_2_SO_4_ was added to the pellet, and kept for 2 h at 100°C. The mixture was filtered through a 0.22 μm filter (Nylon-66, MDI Membrane Technologies, India) and separated in an HPLC (Agilent Technologies, United States) for the measurement of released glucose. Aminex HPX 87H column was used and 5 mM sulfuric acid was employed as the mobile phase at a flow rate of 0.3 mL min^–1^.

### Measurement of Photosynthetic Pigments

The photosynthetic pigments chlorophyll *a* and the carotenoids were measured as described in Gupta et al. (2020). One milliliter culture of cells resuspended at OD730 of ∼2 was centrifuged in a 1.5 mL microcentrifuge tube at 4,000 × *g* for 5 min. One milliliter ice-cold methanol (from Sigma-Aldrich, United States) was added to the pellet and mixed well. The tubes were then kept on ice in dark for 1 h to complete extraction of photosynthetic pigments. The tubes were then centrifuged at 4°C for 7 min at 21,000 × *g*. The resulting supernatant was analyzed spectrophotometrically at specific wavelengths (Lichtenthaler, 1987; Wellburn, 1994).

### Statistical Analysis

Experiments were conducted in biological triplicates. Two-way ANOVA along with Tukey test for pairwise comparison was used for statistical analysis (SigmaPlot version 12.5, Systat Software Inc.).

## Results

### Screening and Confirmation of Transformants by Genomic DNA PCR, qRT-PCR and Western Blotting

Table 1 shows the list of the transformants generated.

**TABLE 1:** Showing the strains and plasmids used in our study.

**Strain**	**Description**
PCC 7002	Wild type *Synechococcus* sp. PCC 7002
A	7002 cells overexpressing SbtA under promoter P_cpcB_ and RBS1
B	7002 cells overexpressing SbtA under promoter P_cpcB__560_ and RBS1
C	7002 cells overexpressing SbtA under promoter P_rbL__2A_ and RBS1
D	7002 cells overexpressing SbtA under promoter P_cpcB_ and RBS2
E	7002 cells overexpressing SbtA under promoter P_cpcB__560_ and RBS2
F	7002 cells overexpressing SbtA under promoter P_rbL__2A_ and RBS2

**Plasmid**	**Description**

pSK +	pBlueScript II SK(+) as backbone vector
pA	Plasmid containing *sbtA* gene cassette, with promoter P_cpcB_ and RBS1
pB	Plasmid containing *sbtA* gene cassette, with promoter P_cpcB__560_ and RBS1
pC	Plasmid containing *sbtA* gene cassette, with promoter P_rbcL__2A_ and RBS1
pD	Plasmid containing *sbtA* gene cassette, with promoter P_cpcB_ and RBS2
pE	Plasmid containing *sbtA* gene cassette, with promoter P_cpcB__560_ and RBS2
pF	Plasmid containing *sbtA* gene cassette, with promoter P_rbcL__2A_ and RBS2

**FIGURE 2:**
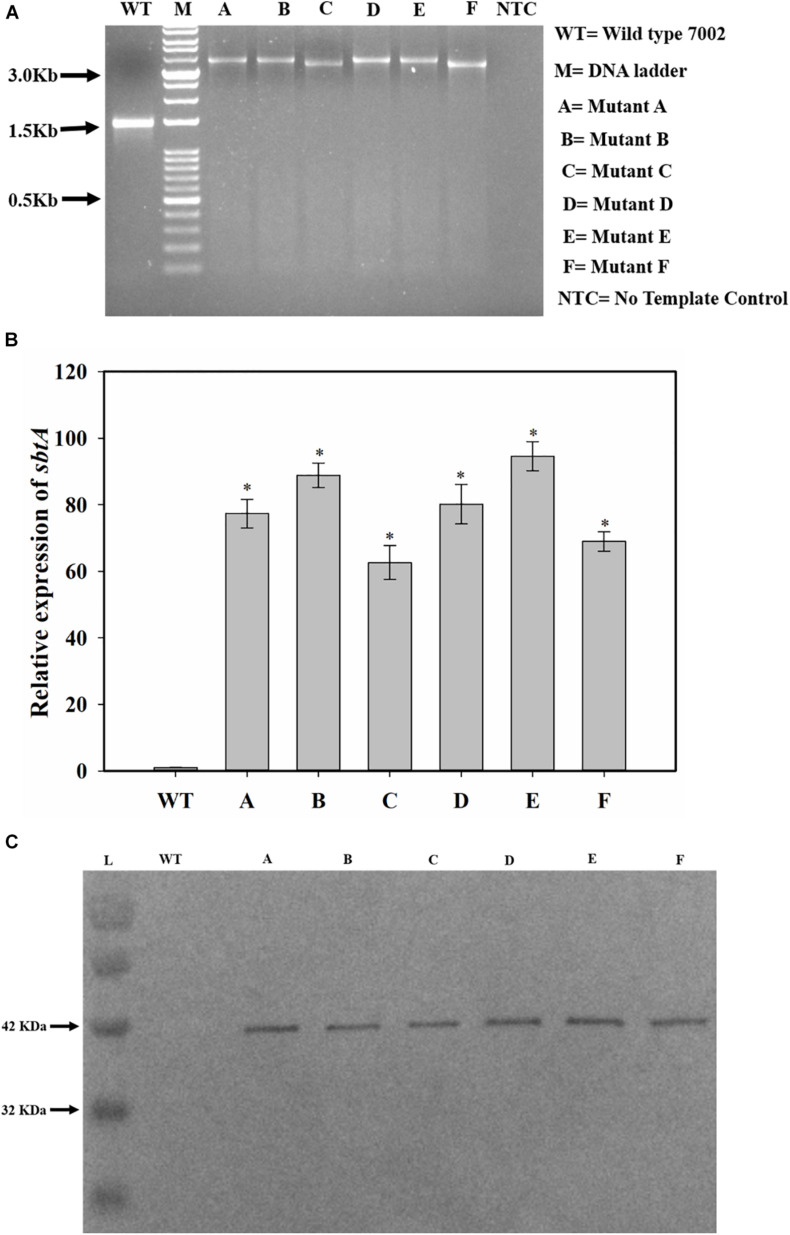
Screening and confirmation of transformants. **(A)** Screening for integration of gene cassette by genomic DNA PCR. *sbtA* gene constructs with different promoters and RBS, containing kanamycin resistance gene as the selection marker were cloned between NSI and NSII in PCC 7002 cells by homologous recombination. The whole insert was PCR amplified using forward primer for NSI and reverse primer for NSII. M is DNA molecular weight marker. Lanes WT, A, B, C, D, E, and F represent the PCR product upon using the genomic DNA from PCC 7002, A, B, C, D, E, and F cells, respectively, as the template. NTC is the no template control in which no DNA template was added. **(B)** Measurement of relative levels of *sbtA* mRNA in the strains A to F by qRT-PCR, n = 3. *p < 0.05 compared to WT PCC 7002 cells. **(C)** Expression of His-tagged SbtA protein in different strains was measured by Western blotting. L = Protein molecular weight marker. Lanes WT, A, B, C, D, E, and F represent proteins extracted from WT PCC 7002, A, B, C, D, E, and F cells, respectively.

NSI to NSII sequence was PCR amplified from the genomic DNA of the WT and transformed PCC 7002 cells. The PCR product size was larger for the transformed cells than for the PCC 7002 cells, indicating integration of the gene cassette in genomic DNA within the neutral sites (Figure 2A). The size of PCR product from the transformants C and F is smaller because the P_rbcL__2A_ promoter is about 300 bases smaller than the other 2 promoters. Transformant F has also been reported our earlier study (Gupta et al., 2020). The expression levels of SbtA were also determined through qRT-PCR. While all the transformants had 60–90-fold higher SbtA levels than WT, transformants C and F had lower levels compared to other transformants (Figure 2B). Relative expression levels of genetically engineered strains A, B, C, D, E, and F were found to be 77.4 ± 4.3, 88.9 ± 3.7, 62.6 ± 5.1, 80.2 ± 5.9, 94.6 ± 4.4, and 68.9 ± 2.9, respectively. All the transformants expressed the His-tagged SbtA protein (Figure 2C).

### Growth of Wild Type and Transformed Cells at Different CO_2_ Concentrations

**FIGURE 3:**
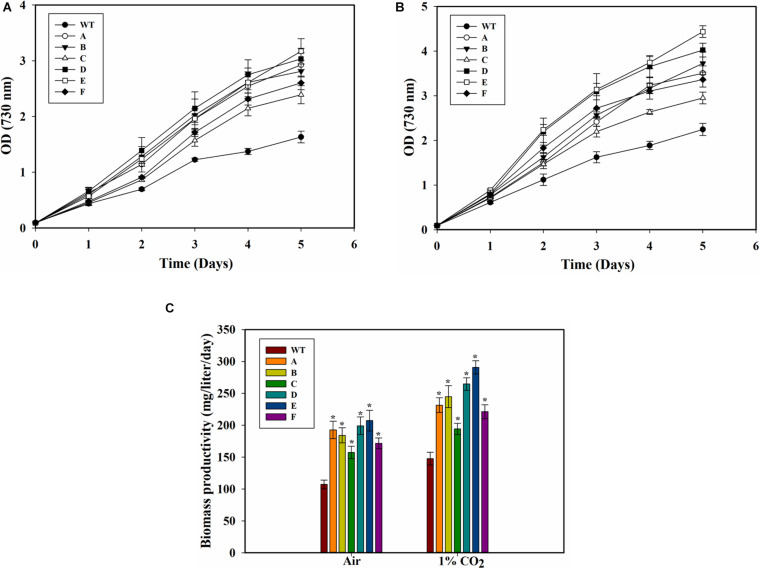
Growth of WT PCC 7002 and transformants. **(A)** Growth with air bubbling. *p < 0.05 for all strains A to F compared to WT PCC 7002 cells for the same day. **(B)** Growth with 1% CO_2_ bubbling. *p < 0.05 compared to WT PCC 7002 cells for the same day. **(C)** Biomass productivity with bubbling of air and 1% CO_2_ after 5 days of growth. *p < 0.05 compared to WT PCC 7002 cells for the same CO_2_ level. *n* = 3 for all the experiments.

The SbtA-overexpressing transformed cells with different promoters and RBSs exhibited significant improvement in growth as compared to the WT PCC 7002 cells when the cultures were bubbled either with air or 1% CO_2_ (Figures 3A,B). Importantly, the cell density (OD730) of all the transformants with air bubbling was higher than that of the WT PCC 7002 cells with 1% CO_2_ bubbling. There was also a significant difference in growth among some of the transformants with either 1% CO_2_ or air bubbling. When cultured either with air bubbling or 1% CO_2_ bubbling, the transformant E (promoter P_cpcB__560_ and RBS2) showed highest difference in growth compared to the WT PCC 7002 cells, with about 90% higher OD with air or 1% CO_2_ bubbling (Figures 3A,B). Transformant C (promoter P_rbcL__2A_ with RBS1) showed the least, nonetheless significant, difference in growth compared to the WT cells.

Biomass productivity of the WT PCC 7002 cells increased with 1% CO_2_ bubbling, with values of 107.6 ± 5.7 and 148.2 ± 7.1 mg⋅L^–1^d^–1^ on air and 1% CO_2_, respectively (Figure 3C). The transformants showed greater biomass productivity compared to the WT PCC 7002 in both air and 1% CO_2_ bubbled cultures. Strain E (the SbtA transformant with P_cpcB__560_ and RBS2) had the highest biomass productivity of 207 ± 16 mg^TN^L^–1^⋅day^–1^ and 290 ± 10 mg⋅L^–1^⋅day^–1^ with bubbling of air and 1% CO_2_, respectively. Among the transformants, strain C had the lowest biomass productivity of about 157 ± 9.6 mg⋅L^–1^⋅d^–1^ with air and 194 ± 8.8 mg⋅L^–1^⋅d^–1^ with 1% CO_2_, although these values are still significantly higher than the WT PCC 7002 cells grown under similar conditions.

### Glycogen Content and Productivity

**FIGURE 4:**
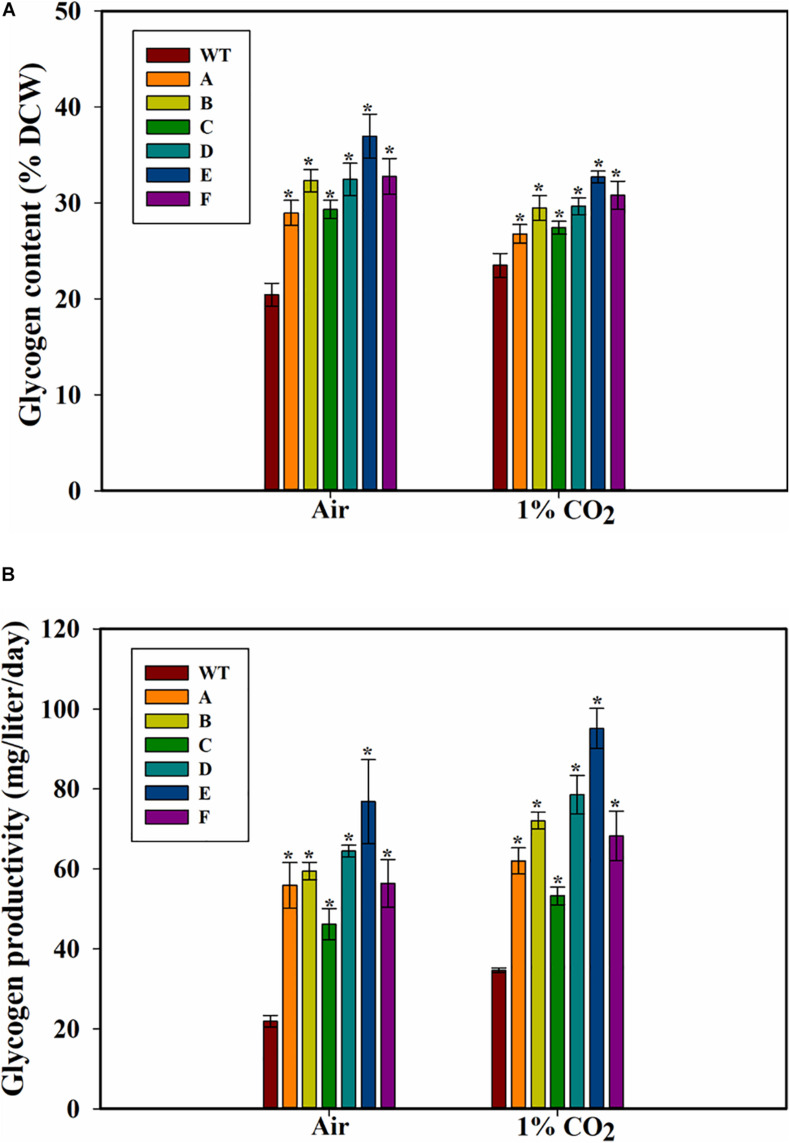
Glycogen content and productivity of the cells. **(A)** Intracellular glycogen content, **(B)** glycogen productivity of WT PCC 7002 and transformants with bubbling of air or 1% CO_2_, measured after 5 days of growth. *p < 0.05 compared to WT PCC 7002 cells grown at the same CO_2_ concentrations. *n* = 3.

Because glycogen is a major storage polysaccharide of the cyanobacterial cells, the glycogen content of the cells was also measured. Higher glycogen content was found in all the SbtA-overexpressing strains compared to WT PCC 7002 cells. The highest glycogen levels in strain E, was observed when the cells were cultured either with air or 1% CO_2_ bubbling (Figure 4A). After 5 days of growth on air bubbling, the glycogen content was up to 36.9 ± 2.3% (of DCW) in the strain E while it was only 20 ± 1% (of DCW) in WT PCC 7002 cells (Figure 4A). However, unlike the significant increase in growth observed when the cells were bubbled with 1% CO_2_ compared to air bubbling, there was not a significant change in glycogen content of the cells when bubbled with 1% CO_2_ compared to when bubbled with air.

The glycogen productivities of the transformants were significantly higher than that of WT PCC 7002 cells whether with air bubbling or with 1% CO_2_ bubbling (Figure 4B). Strain E grown on air showed the greatest increase in glycogen productivity of about 2.5-fold compared to WT PCC 7002 (Figure 4B) while the transformant C showed the least increase in productivity (∼60% increase when grown on 1% CO_2_). Interestingly, while the WT PCC 7002 cells showed 1.5-fold glycogen productivity when grown on 1% CO_2_ compared to air, the transformants showed only a slight increase in productivity at 1% CO_2_ compared to air. Thus, the increase in glycogen productivity in 1% CO_2_ is primarily due to the faster growth of cells.

### Pigment Content of the Transformed Cells

**FIGURE 5:**
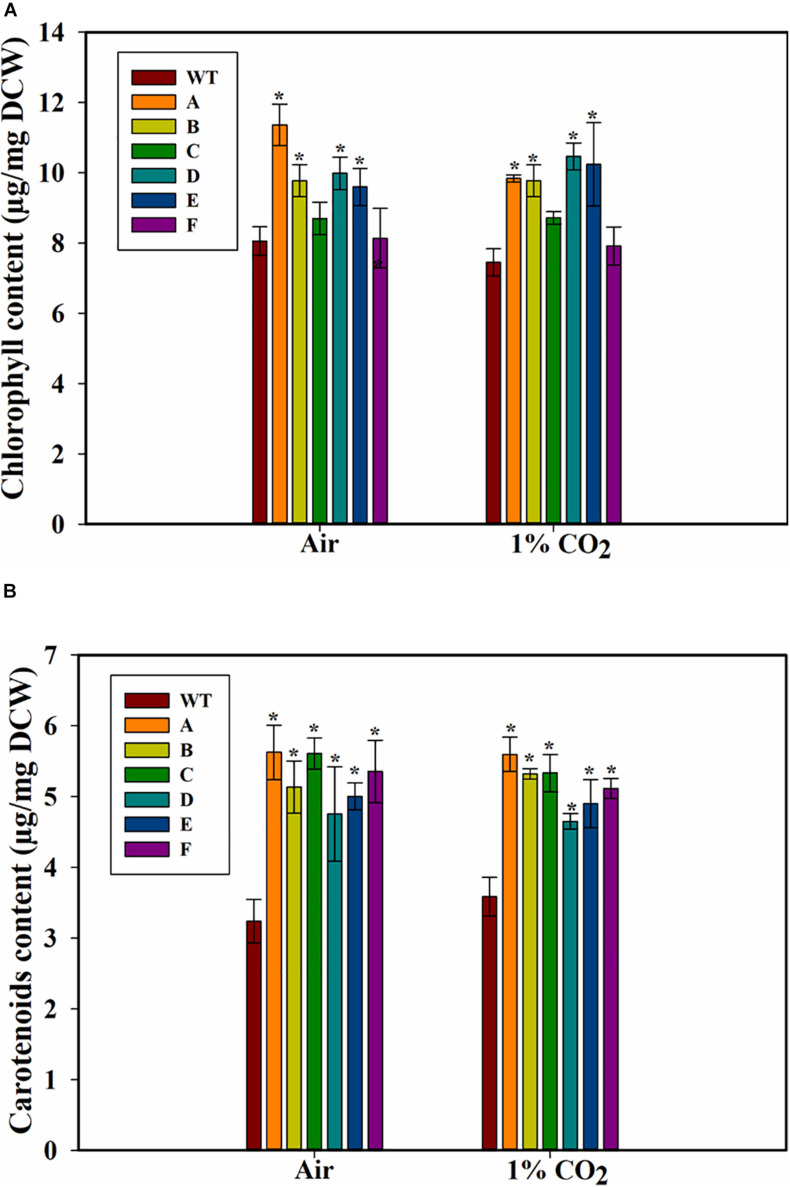
Pigment content of wild type (PCC 7002) and transformants overexpressing SbtA with different promoters and RBSs (strains A to F) after 5 days of growth. **(A)** Chlorophyll content, and **(B)** carotenoids content, with the bubbling of air or 1% CO_2_. *p < 0.05 compared to WT PCC 7002 cells grown under similar CO_2_ level, *n* = 3.

Because growth can also be affected by chlorophyll levels, we measured the cellular chlorophyll contents of the WT PCC 7002 and transformants. The strain A showed the highest amount of chl *a* among all the strains when cultured on air (Figure 5A). Strains C and F, when grown on air or 1% CO_2_, did not have higher chlorophyll levels compared to WT PCC 7002 cells. Transformants A, B, D, and E had similar levels of chlorophyll, which was higher than that in the PCC 7002 cells. Also, no effect of higher CO_2_ concentration on chlorophyll levels was observed.

A significant increase in the amount of carotenoids was observed in the transformants both either with air and 1% CO_2_, and an increase of up to ~70% in the content of carotenoids (accessory light harvesting pigments) was observed in the transformed cells when grown in presence of air (Figure 5B). Interestingly, unlike the response of chlorophyll content, all the transformants had higher carotenoid content.

## Discussion

Glycogen is a potential fermentation feedstock (Möllers et al., 2014). Also, glycogen is more water soluble and can be more easily hydrolyzed to simple sugars compared to other storage polysaccharides like starch because it is more branched (Ball et al., 2011). Our study shows that improving Carbon transport through overexpression of SbtA can increase cyanobacterial biomass and glycogen productivity. The combinatorial effects of choice of RBS and promoters on the expression of a membrane protein, and its physiological effects, are presented for the first time. A correlation between SbtA expression and growth rate was observed among the transformants. The transformant E with P_cpcB__560_ and RBS2 showed the highest growth and the glycogen content. An interesting outcome was that all the transformants grown on air had higher glycogen productivity than WT PCC 7002 cells grown on 1% CO_2_ with the transformant E showing up to 2.5-fold increase. The increased glycogen productivity in 1% CO_2_ grown cells due to increased biomass accumulation and not due to increased cellular glycogen levels.

Among the promoters tested, P_cpcB__560_ had the strongest effect, in agreement with its super-strong activity due to the presence of multiple TFBS in its sequence (Zhou et al., 2014). This is in spite of previous reports that P_cpcB_ is inhibited under strong light conditions (Sengupta et al., 2020a). The strong and continuous illumination was chosen in our study because previous studies, including our own preliminary studies, have shown that it leads to higher biomass accumulation for the WT PCC 7002 cells. P_rbcL__2A_ had a slightly lesser effect on SbtA expression, growth and glycogen accumulation compared to the P_cpcB_ promoters. Our study shows that even under air bubbling and high illumination, very similar to the “optimal” conditions for high P_rbcL_ activity (Sengupta et al., 2020a) P_cpcB__560_ has higher activity compared to PrbcL2A. Promoter activity may also be affected by the length of the promoter chosen. We chose lengths of 272 bp for P_rbcL__2A_, 589 for P_cpcB_ and 560 bp of P_cpcB__560_, respectively based the high activity in PCC 7002 reported in a previous study (Huang et al., 2010; Zhou et al., 2014).

Another factor we tested was the choice of RBSs. AGGAGG has been suggested as the common RBS for PCC 7002 (Gordon and Pfleger, 2018). A previous study (Markley et al., 2015) had studied 11 different RBS sequences in PCC 7002 cells and reported highest eYFP levels when RBS2 (AGGAGA) was used. The levels for AGGAGA were about twice than that for AGGAGG. As such, the choice of RBS had only a minor effect on SbtA expression, growth and glycogen productivity. Therefore, we do not expect any significant improvement when using the common RBS (AGGAGG) of PCC 7002 cells.

Our study shows that overexpression of SbtA can change the cellular chlorophyll and carotenoids content. One common response was that chlorophyll levels are not affected by the concentration of the CO_2_ bubbled, i.e., either with air and 1% CO_2_ bubbling, even though biomass productivity of cells grown on 1% CO_2_ was 30–45% higher than those grown on air. This indicates that the basal chlorophyll level is enough to support elevated growth. One possible reason for increased carotenoids in the transformants could be that due to their faster growth, the transformants experience nutrient stress before the WT PCC 7002 cells. Nutrient stress is associated with higher carotenoid levels (Wang and Peng, 2008; Lemoine and Schoefs, 2010; Minhas et al., 2016). Carotenoids also play an important role in protecting the cells from photo-oxidative damage (Demmig-Adams and Adams, 2002). However, our cultures were exposed to similar light intensity and this cannot explain the variation observed. The underlying mechanisms of regulation of the pigments are not clear and will require further investigation which is out of the scope of current study. Of interest will be to identify the metabolomic changes in the transformants (Hasunuma et al., 2013; Schwarz et al., 2013; Behrendt et al., 2018; Jaiswal and Wangikar, 2020) and how the variation in SbtA levels alter the bicarbonate uptake rates and intracellular labeling rates through 13C-metabolic labeling studies (Alagesan et al., 2013; Prasannan et al., 2019) as well as the proteomic changes in the transformants.

Overall, our results show that choice of a strong RBS and promoter while overexpressing the bicarbonate transporter SbtA can have significant impact on cellular physiology. Our results suggests that engineering high activity, regulatable promoters will help improving protein expression and hence product formation. Our results also show a complex regulation of cellular pigments which warrant further studies.

## Conclusion

Our results show significant improvements in growth and glycogen content of the cells overexpressing the bicarbonate transporter SbtA. Additionally, the promoter as well as the RBS chosen to overexpress SbtA was shown to impact glycogen productivity. A combination of a strong promoter and a strong RBS led to a transformant with glycogen productivity that was about 2.7–3.5-fold that of the WT PCC 7002 cells depending on whether 1% CO_2_ or air was used for growth. This increase was due to higher biomass as well as increased glycogen content in the transformants.

## Data Availability Statement

The original contributions presented in the study are included in the article/Supplementary Material, further inquiries can be directed to the corresponding author/s.

## Author Contributions

SS and JG designed the study and wrote the manuscript together. JG conducted all the experimental work which was supervised by SS. Both authors contributed to the article and approved the submitted version.

## Conflict of Interest

The authors declare that the research was conducted in the absence of any commercial or financial relationships that could be construed as a potential conflict of interest.

## Supplementary Material

Supplementary Table 1 The raw data used to construct various figures.

Supplementary Data Sheet 1 The sequence of sbtA gene from PCC 7002, promoters sequences, the RBS sequences used in the study, genes and their sources, primers used for cloning, and RT-PCR primers used to check expression. It also includes maps of various vectors.
