# Supplementary material for: The Effect of Promoter and RBS Combination on the Growth and Glycogen Productivity of Sodium-Dependent Bicarbonate Transporter (SbtA) Overexpressing Synechococcus sp. PCC 7002 Cells
Source: Front Microbiol. 2021 Apr 13;12:607411. doi: 10.3389/fmicb.2021.607411 (PMC8076525; doi:10.3389/fmicb.2021.607411)
Supplement: Supplementary Data Sheet 1 — The sequence of sbtA gene from PCC 7002, promoters sequences, the RBS sequences used in the study, genes and their sources, primers used for cloning, and RT-PCR primers used to check expression. It also includes maps of various vectors. [file Data_Sheet_1.docx]

**Supplementary data for article**

The effect of promoter and RBS combination on the growth and glycogen productivity of sodium-dependent bicarbonate transporter (SbtA) overexpressing *Synechococcus* *sp.* PCC 7002 cells

**Figure S1. Sequence of the *sbtA* gene (SYNPCC7002_A0470).**

GTGGATTTTTTGTCCGATTTTTTGACGAAATTTGGGTCGCAGTTGCAGTCCCCGACGCTC

GGCTTTTTAATTGGCGGTATTGTCATTGCCGCCTTCGGTAGCCGACTTACAATCCCCGAT

GCAGTGTATAAGTTCATCGTTTTTATGCTGCTCATCAAAGTCGGTTTGAGCGGCGGTATT

GCGATTCGTAATACCAATATCACGGAGATGCTCTTGCCTGCGTTATTTGCTGTGCTCATG

GGCATTCTGATCGTTTTTATTGGGCGTTTTACCTTAGCAAAGCTGCCAGGTATTAGGACC

GTAGATGCAGTGGCAACTGCCGGCTTGTTTGGGGCAGTGAGTGGTTCGACCCTTGCTGCT

GGAATCACGGTTATGGAAGGGCAAGGTGTTTTCTACGAACCTTGGGCAGCGGCACTTTAT

CCTTTTATGGATATTCCCGCCCTGGTGACAGCGATTGTTGTAGCTAGTCTTTATAAGAGC

AAGCAGCGCGAGGTTGAAGCCGATGATTTCAGCAAACAACCCGTTGCCGCTGGTGAATAT

TCTGGTGAACCTGTTTATCCCACCACGAGGCAGGAATATCTGGGTCAAAAGCGTGGTAAG

GCTACTAATCGGGTTGAAATTTGGCCCATTGTTAAGGAAAGTCTCCAGGGTTCTGCCCTA

TCAGCATTGTTGCTCGGTCTTGCTCTCGGTTTGTTGACTCGGCCAGAAAGTGTCTTTGAA

AGTTTCTATGAGCCCCTCTTCCGTGGTTTTCTTTCGATTTTGATGCTGGTGATGGGGATG

GAAGCTTGGTCTAGGCTTGGGGAACTGCGCAAAGTTGCTCAATGGTACGCTGTCTATGCG

TTTATTGCGCCGCTACTCCATGGGTTTATTGCCTTCGGTCTCGGCATGATCGCCCACTAT

GTTACAGGGTTCAGTCCTGGTGGTGTTGCCCTCTTAGCGATTATTGCGGCGTCTAGTTCG

GACATCTCTGGGCCGCCTACTTTACGGGCTGGGATTCCGTCGGCTAATCCTTCTGCTTAT

ATCGGTTCGTCTACGGCCATCGGTACCCCCGTGGCGATCGCCATCGGCATACCACTTTTT

ATCGGCCTTGCCCAAGCAACCATGGGTGGCTGA

**Figure S2. Promoter sequences (Note: these are the native promoter sequences. Please see Figure S3 for the part of promoter sequences used for cloning)**

**Figure S2.1. Promoter P_cpcB_ sequence (589 base pairs) (from *Synechocystis* sp. PCC 6803)**

GTTATAAAATAAACTTAACAAATCTATACCCACCTGTAGAGAAGAGTCCCTGAATATCAAAATGGTGGGATAAAAAGCTCAAAAAGGAAAGTAGGCTGTGGTTCCCTAGGCAACAGTCTTCCCTACCCCACTGGAAACTAAAAAAACGAGAAAAGTTCGCACCGAACATCAATTGCATAATTTTAGCCCTAAAACATAAGCTGAACGAAACTGGTTGTCTTCCCTTCCCAATCCAGGACAATCTGAGAATCCCCTGCAACATTACTTAACAAAAAAGCAGGAATAAAATTAACAAGATGTAACAGACATAAGTCCCATCACCGTTGTATAAAGTTAACTGTGGGATTGCAAAAGCATTCAAGCCTAGGCGCTGAGCTGTTTGAGCATCCCGGTGGCCCTTGTCGCTGCCTCCGTGTTTCTCCCTGGATTTATTTAGGTAATATCTCTCATAAATCCCCGGGTAGTTAACGAAAGTTAATGGAGATCAGTAACAATAACTCTAGGGTCATTACTTTGGACTCCCTCAGTTTATCCGGGGGAATTGTGTTTAAGAAAATCCCAACTCATAAAGTCAAGTAGGAGATTAATT

**Figure S2.2. Promoter P_cpcB560_ sequence (560 base pairs) (from *Synechocystis* sp. PCC 6803)**

ACCTGTAGAGAAGAGTCCCTGAATATCAAAATGGTGGGATAAAAAGCTCAAAAAGGAAAGTAGGCTGTGGTTCCCTAGGCAACAGTCTTCCCTACCCCACTGGAAACTAAAAAAACGAGAAAAGTTCGCACCGAACATCAATTGCATAATTTTAGCCCTAAAACATAAGCTGAACGAAACTGGTTGTCTTCCCTTCCCAATCCAGGACAATCTGAGAATCCCCTGCAACATTACTTAACAAAAAAGCAGGAATAAAATTAACAAGATGTAACAGACATAAGTCCCATCACCGTTGTATAAAGTTAACTGTGGGATTGCAAAAGCATTCAAGCCTAGGCGCTGAGCTGTTTGAGCATCCCGGTGGCCCTTGTCGCTGCCTCCGTGTTTCTCCCTGGATTTATTTAGGTAATATCTCTCATAAATCCCCGGGTAGTTAACGAAAGTTAATGGAGATCAGTAACAATAACTCTAGGGTCATTACTTTGGACTCCCTCAGTTTATCCGGGGGAATTGTGTTTAAGAAAATCCCAACTCATAAAGTCAAGTAGGAGATTAATTCA

**Figure S2.3. Promoter P_rbcL2A_ sequence (272 base pairs) (from *Synechocystis* sp. PCC 6803)**

TCACCATTTGGACAAAACATCAGCAATTCTAATTAGAAAGTCCAAAAATTGTAATTTAAAAAACAGTCAATGGAGAGCATTGCCATAAGTAAAGGCATCCCCTGCGTGATAAGATTACCTTCAGAAAACAGATAGTTGCTGGGTTATCGCAGATTTTTCTCGCAACCAAATAACTGTAAATAATAACTGTCTCTGGGGCGACGGTAGGCTTTATATTGCCAAATTTCGCCCGTGGGAGAAAGCTAGGCTATTCAATGTTTAAAGAGGAGAAA

**Figure S3. Promoter stretches used for cloning**

**Figure S3.1. Promoter P_cpcB_ stretch (577 base pairs) (from *Synechocystis* sp. PCC 6803)**

GTTATAAAATAAACTTAACAAATCTATACCCACCTGTAGAGAAGAGTCCCTGAATATCAAAATGGTGGGATAAAAAGCTCAAAAAGGAAAGTAGGCTGTGGTTCCCTAGGCAACAGTCTTCCCTACCCCACTGGAAACTAAAAAAACGAGAAAAGTTCGCACCGAACATCAATTGCATAATTTTAGCCCTAAAACATAAGCTGAACGAAACTGGTTGTCTTCCCTTCCCAATCCAGGACAATCTGAGAATCCCCTGCAACATTACTTAACAAAAAAGCAGGAATAAAATTAACAAGATGTAACAGACATAAGTCCCATCACCGTTGTATAAAGTTAACTGTGGGATTGCAAAAGCATTCAAGCCTAGGCGCTGAGCTGTTTGAGCATCCCGGTGGCCCTTGTCGCTGCCTCCGTGTTTCTCCCTGGATTTATTTAGGTAATATCTCTCATAAATCCCCGGGTAGTTAACGAAAGTTAATGGAGATCAGTAACAATAACTCTAGGGTCATTACTTTGGACTCCCTCAGTTTATCCGGGGGAATTGTGTTTAAGAAAATCCCAACTCATAAAGTCAAGT

**Figure S3.2. Promoter P_cpcB560_ stretch (547 base pairs) (from *Synechocystis* sp. PCC 6803)**

ACCTGTAGAGAAGAGTCCCTGAATATCAAAATGGTGGGATAAAAAGCTCAAAAAGGAAAGTAGGCTGTGGTTCCCTAGGCAACAGTCTTCCCTACCCCACTGGAAACTAAAAAAACGAGAAAAGTTCGCACCGAACATCAATTGCATAATTTTAGCCCTAAAACATAAGCTGAACGAAACTGGTTGTCTTCCCTTCCCAATCCAGGACAATCTGAGAATCCCCTGCAACATTACTTAACAAAAAAGCAGGAATAAAATTAACAAGATGTAACAGACATAAGTCCCATCACCGTTGTATAAAGTTAACTGTGGGATTGCAAAAGCATTCAAGCCTAGGCGCTGAGCTGTTTGAGCATCCCGGTGGCCCTTGTCGCTGCCTCCGTGTTTCTCCCTGGATTTATTTAGGTAATATCTCTCATAAATCCCCGGGTAGTTAACGAAAGTTAATGGAGATCAGTAACAATAACTCTAGGGTCATTACTTTGGACTCCCTCAGTTTATCCGGGGGAATTGTGTTTAAGAAAATCCCAACTCATAAAGTCAAGTA

**Figure S3.3. Promoter P_rbcL2A_ stretch (262 base pairs) (from Synechocystis sp. PCC 6803)**

TCACCATTTGGACAAAACATCAGCAATTCTAATTAGAAAGTCCAAAAATTGTAATTTAAAAAACAGTCAATGGAGAGCATTGCCATAAGTAAAGGCATCCCCTGCGTGATAAGATTACCTTCAGAAAACAGATAGTTGCTGGGTTATCGCAGATTTTTCTCGCAACCAAATAACTGTAAATAATAACTGTCTCTGGGGCGACGGTAGGCTTTATATTGCCAAATTTCGCCCGTGGGAGAAAGCTAGGCTATTCAATGTTTAA

**Table S1. Genes and sources**

| **Gene** | **Source** |
| --- | --- |
| NSI/SYNPCC7002_A0935 | *S.*7002 genomic DNA |
| P_cpcB_ | *S.*6803 genomic DNA |
| P_cpcB560_ | *S.*6803 genomic DNA |
| P_rbcL2A_ | *S.*6803 genomic DNA |
| *sbtA* | *S.*7002 genomic DNA |
| *kanR2* | Vector pET-28a(+) |
| *GroEL* Ter | *S.*7002 genomic DNA |
| NSII/ SYNPCC7002_A0936 | *S.*7002 genomic DNA |

**Table S2. Primers Sequences for *sbtA* cassette**

| **Primer Name** | **Sequence** |
| --- | --- |
| **NSI-FP** | CAGTACTCGAGATGTTTGGTCGAAAATTCTTCACACT |
| **NSI-RP** | CAGTAGTCGACTTACTCAGTTTTTAAGTAATTAGCAGA |
| **P_cpcB_-FP** | CAGTAGTCGACGTTATAAAATAAACTTAACAAATCTATAC |
| **P_cpcB_-RP** | CAGTAGATATCACTTGACTTTATGAGTTGGGATTTTC |
| **P_cpcB560_-FP** | CAGTAGTCGACACCTGTAGAGAAGAGTCCCTGAA |
| **P_cpcB560_-RP** | CAGTAGATATCTACTTGACTTTATGAGTTGGGATTTT |
| **P_rbcL2A_-FP** | CAGTAGTCGACTCACCATTTGGACAAAACATCAGC |
| **P_rbcL2A_-RP** | CAGTAGATATCTTAAACATTGAATAGCCTAGCTTTCT |
| ***sbtA*-FP1*** | CAGTAGATATC**GGAGGA**AAACCGCGTGGATTTTTTGTCCGATT |
| ***sbtA*-FP2*** | CAGTAGATATC**AGGAGA**AAACCGCGTGGATTTTTTGTCCGATT |
| ***sbtA*-RP** | CAGTAGAATTCTCAGTGGTGGTGGTGGTGGTGGCCACCCATGGTTGCT |
| ***kan*-FP** | CAGTAGAATTCGGAGGAAAACCGCATGCACCACCACCACCACCACATGAGCCATATTCAACGGGAAAC |
| ***kan*-RP** | CAGTAGGATCCTTAGAAAAACTCATCGAGCATCAAA |
| **Ter-FP** | CAGTAGGATCCGGTTTAGTGACCGACTAACACTTT |
| **Ter-RP** | CAGTAACTAGTAAACGAAAGAGAGCTTAGCAAGTG |
| **NSII-FP** | CAGTAACTAGTCTATTTTATCTCGTTTTCTTCCCAG |
| **NSII-RP** | CAGTAGAGCTCATGAATAAACTGTTTACAGCAGCC |

*** The RBS sequences are shown in bold.**

**Table S3. RT-PCR Primers**

| **Primer Name** | **Sequence** |
| --- | --- |
| **rt*ppC*-FP** | CATCGGCCGCTTCTACTTTAT |
| **rt*ppC*-RP** | ATCGTTACCGCCAAGAACCC |
| **rt*sbtA*-FP** | CGATTTTGATGCTGGTGATG |
| **rt*sbtA*-RP** | GCCGCAATAATCGCTAAGAG |

**A**

**B**

**C**

**D**

**E**

**F**

**G**

**Figure S4:** Vectors maps viz. (A) pBluescript SK II (+), used as cloning vector. Other vectors carry the *sbtA* gene along with respective promoters and RBSs, and other parts of gene cassettes viz. neutral site I, *kanR*, Terminator and neutral site II (B) pA contains promoter PcpcB and RBS1; (C) pB contains promoter PcpcB560 and RBS1; (D) pC contains promoter PrbcL2A and RBS1; (E) pD contains promoter PcpcB and RBS2; (F) pE contains promoter PcpcB560 and RBS2 and (G) pF contains promoter PrbcL2A and RBS2.
